# Consequences of maternal mortality on infant and child survival: a 25-year longitudinal analysis in Butajira Ethiopia (1987-2011)

**DOI:** 10.1186/1742-4755-12-S1-S4

**Published:** 2015-05-06

**Authors:** Corrina Moucheraud, Alemayehu Worku, Mitike Molla, Jocelyn E Finlay, Jennifer Leaning, Alicia Ely Yamin

**Affiliations:** 1 Harvard T.H. Chan School of Public Health, Boston MA, USA; 2 School of Public Health, College of Health Sciences, Addis Ababa University, Addis Ababa, Ethiopia

**Keywords:** Maternal mortality, infant mortality, child survival, Ethiopia

## Abstract

**Background:**

Maternal mortality remains the leading cause of death and disability for reproductive-age women in resource-poor countries. The impact of a mother’s death on child outcomes is likely severe but has not been well quantified. This analysis examines survival outcomes for children whose mothers die during or shortly after childbirth in Butajira, Ethiopia.

**Methods:**

This study uses data from the Butajira Health and Demographic Surveillance System (HDSS) site. Child outcomes were assessed using statistical tests to compare survival trajectories and age-specific mortality rates for children who did and did not experience a maternal death. The analyses leveraged the advantages of a large, long-term longitudinal dataset with a high frequency of data collection; but used a strict date-based method to code maternal deaths (as occurring within 42 or 365 days of childbirth), which may be subject to misclassification or recall bias.

**Results:**

Between 1987 and 2011, there were 18189 live births to 5119 mothers; and 73 mothers of 78 children died within the first year of their child’s life, with 45% of these (n=30) classified as maternal deaths due to women dying within 42 days of childbirth. Among the maternal deaths, 81% of these infants also died. Children who experienced a maternal death within 42 days of their birth faced 46 times greater risk of dying within one month when compared to babies whose mothers survived (95% confidence interval 25.84-81.92; or adjusted ratio, 57.24 with confidence interval 25.31-129.49).

**Conclusions:**

When a woman in this study population experienced a maternal death, her infant was much more likely to die than to survive—and the survival trajectory of these children is far worse than those of mothers who do not die postpartum. This highlights the importance of investigating how clinical care and socio-economic support programs can better address the needs of orphans, both throughout the intra- and post-partum periods as well as over the life course.

## Background

Maternal mortality is a leading cause of death and disability for adult women worldwide, responsible for an estimated 289,000 deaths in 2013 [1]. It represents true excess burden of disease since the overwhelming majority of maternal deaths are due to preventable causes; and could be treated with well-understood interventions that have long been available in the global North. Maternal mortality highlights large inequalities between and within countries; the maternal mortality rate in resource-poor countries is 15 times higher than that in wealthy nations, and within countries, the poorest women see the greatest risk of dying during pregnancy or childbirth [2]. Reducing maternal mortality ratios (MMRs) by 75% from 1990 levels was therefore included in the United Nations’ Millennium Development Goals, as integral to reducing global poverty. Less well-characterized, however, are the short- and long-term consequences of maternal deaths on children, families and communities. Often a maternal death can have spillover effects onto child health, via obstetric complications, infant feeding behaviors, and care for orphans. It thus is critically important to look beyond MMRs to fully characterize the harm caused by the loss of a mother.

There are a number of mechanisms through which a maternal death may affect outcomes for infants and children. The main direct causes of maternal mortality—obstetric complications such as eclampsia, sepsis, obstructed labor and hemorrhage—can also put neonates at increased risk of death [3-5]. If the infant survives birth but the mother does not, the resulting lack of nutritional support from breastfeeding leaves the baby vulnerable to malnutrition, which can itself be fatal or may increase the risk of disease or death from infection [6-8]. Older siblings also may suffer in many ways without maternal care: among orphans, the risk of child labor [9,10], poor learning outcomes and lower educational attainment [11], and disrupted living arrangements [12] can impose trauma that has detrimental impacts on health and well-being. Qualitative research from rural Tanzania found that orphans of women who died of maternal causes—girls in particular—were likely to be undernourished in infancy and beyond, face education-related challenges, and receive compromised medical care [13]. This study also found adverse household effects, including economic drains—a finding echoed in a recent study from China, where maternal death was associated with significant household income and expenditure declines [14,15]. Detrimental household economic impacts were also seen in a study from Burkina Faso, where the high and unforeseen expense of emergency obstetric care was reported as difficult to repay, triggering long-term consequences on physical, psychological, social and economic well-being [16].

Infant and child mortality is thus only one adverse outcome associated with maternal death, but it is crucially important. An analysis from Bangladesh found a significantly worse survival trajectory of orphaned children—but cautioned about generalizing the findings, due to contextual factors that may differentially impact orphan survival such as household composition, the role of the father and HIV prevalence [6]. Recent analyses, however, have found similarly negative outcomes in sub-Saharan Africa. A cohort study in Benin found an elevated risk of mortality among infants born to women who experienced serious complications during childbirth (near-miss cases), even in the absence of maternal death [17]. Recent research in Kenya found an elevated mortality rate among babies of women who died after childbirth [18].

This analysis aims to provide evidence about the grave mortality consequences of maternal death on young children in Ethiopia between 1987 and 2011. By using a longitudinal demographic dataset—from a Health and Demographic Surveillance System (HDSS) site, comparable to the methods used in the Ronsmans study cited above [6]—we conducted a full survival analysis for all children of deceased mothers, examining additional outcomes for older children who become orphaned, and applying different definitions of maternal death in order to capture the consequences of a mother’s death immediately after birth, as well as to examine a longer-range effect for late maternal deaths.

## Methods

### Study setting and data collection

This analysis uses a household-level longitudinal dataset, collected by the Butajira Rural Health Programme as a member center and founder in the INDEPTH network of HDSS sites. Butajira is a woreda (district) in south-central Ethiopia, approximately 130 kilometers south of Addis Ababa; the HDSS site consists of both rural- and town-based households. Data collection began in 1987 with monthly visits to approximately 28,000 individuals; the frequency was reduced to quarterly study visits in 2000. Date of closure for the current analysis was mid-2011, by which time the study population had grown to approximately 70,000 individuals. During household visits, study personnel collected information about vital and migratory events: births, deaths, and changes in household composition including marriage, and in- and out-migration. In the event of a parental death, the orphaned children were followed in subsequent rounds of data collection if they relocated within the Butajira study area. All those who moved out of the community were lost to follow-up; although such loss to follow-up is estimated to be low among reproductive-age women in Butajira [unpublished data].

### Variable definition

A subset of the full Butajira HDSS dataset was used for the current analysis: children born to women during the study period (i.e., between January 1987 and June 2011). “Maternal death” was operationalized as a woman’s death within a 42-day window of her most recent childbirth (or within 365 days, using the standard definition for late maternal death). It was necessary to use a date-based method to categorize maternal deaths because there are not reliable cause-of-death data available in the Butajira HDSS dataset. These categorizations were selected to correspond with the WHO definitions of maternal death, although the latter also includes deaths during pregnancy, which was not explored in the main analysis here (due to limitations in these data).

The “index child” was defined as the reference birth for counting these 42 (or 365) days, and “non-index children” were all births prior to the one associated with the maternal death (i.e., elder siblings of the index child). Children’s survival time was calculated as the number of days from their birth until their death or out-migration, or the study period end. A woman’s death during her pregnancy was reported as such by other members of her household during regular data collection.

Household characteristics were recorded only at baseline. For our analysis, women were classified into three wealth groups based on a principal component analysis of household assets (per [19]); the score included: number of rooms in the home, the presence of a separate kitchen, the presence of windows, use of piped or protected well water, use of a functional flush toilet, radio ownership, and a metal (non-thatched) roof.

### Statistical methods

We used Kaplan-Meier survival analysis to calculate cumulative survival probabilities for index children of a maternal death versus those with surviving mothers from birth to 30 days (1 month), 183 days (6 months), 365 days (12 months), 1825 days (5 years) and 3652 days (10 years). Statistically significant differences between these survival functions were assessed using the log-rank test. We used a Poisson regression to compare death rates within each of these age groups for index children of a maternal death and children with a surviving mother; the unadjusted ratios used robust standard errors to account for clustering of births to the same mother, and the adjusted ratios added controls for other important covariates: household wealth, mother’s age, mother’s ever marital status, and mother’s educational attainment. Confidence intervals at the 95% level are reported. All analyses were conducted using Stata 12.1 (StataCorp 2014).

### Ethical clearance

Study protocols were approved by the Harvard T.H. Chan School of Public Health Institutional Review Board and the Addis Ababa University College of Health Sciences Institutional Review Board in Ethiopia.

## Results

Table 1 presents information on the mothers and children analyzed in this study, including classifications of deaths and survival. Between 1987 and 2011, there were 17993 live births to 5084 mothers; 30 mothers of 104 children died within the first 42 days of childbirth.

**Table 1:** Characteristics of mothers and children in the Butajira cohort, 1987-2011

			n	%
**Maternal death within 42 days** n= 30	Index child n= 32	Deceased	26	81.25%
		
		Survived	6	18.75%
	
				
	
	Non-index children n= 72	Deceased	5	6.94%
		
		Survived	67	93.06%

				

**Non-maternal death** n= 335	Children n= 1014	Deceased	45	4.44%
		
		Survived	969	95.56%

				

**Surviving women** n= 4719	Children n= 16875	Deceased	1509	8.94%
		
		Survived	15366	91.06%

Note: The 26 deceased index children include 5 children who died on the day of their birth, and their mother’s death occurred within the following 4 days. All other children classified as deceased in this analysis (i.e., non-index children of maternal deaths and all children of women who died from non-maternal causes) died subsequent to their mother’s death.

Children born to women who died around the time of their childbirth experienced very high mortality: 81% of these children also died. This is significantly higher (p<0.001) than the proportion of deaths among older children of these women (7%). The mortality proportion among children of maternal deaths (combined index and non-index births) is significantly higher (p<0.001) than that among children of women who died non-maternal deaths (4.4%). And among children who experienced death of a mother (maternal or non-maternal causes), more of these children died than children of women who survived (p<0.01), among whom the proportion of deaths is around 9%. Notably, non-index children of maternal deaths do not have a significantly different (p=0.6) probability of death than children whose mothers survived (7% and 9%, respectively).

Characteristics of the study sample are presented in Table 2 for mothers, and Table 3 for children. Women who died during or shortly after childbirth are significantly (p<0.001) more commonly in the poorest wealth groups: 60% of deceased women were in the lowest household asset group (versus 30% of surviving women); note that this is a relative measure (relative asset index to other households in the community). The sex distribution of children is not statistically significantly different between the groups (p=0.3). Women were at increased risk of maternal death at first birth and at high parity: this is reflected in the birth order among index children of maternal deaths, where 34% of index children to maternal deaths were a first birth, and 38% were birth order five and beyond. Birth order and maternal age are likely associated, and this table presents only uncontrolled proportions.

**Table 2:** Characteristics of mothers in the Butajira cohort, 1987-2011

	Maternal death (42 days)	Mother survived
n	30	4719

**Mother’s age at most recent childbirth (years)**		

10-19	2 (6.7%)	254 (5.38%)

20-24	5 (16.7%)	712 (15.09%)

25-29	6 (20.0%)	938 (19.88%)

30-34	8 (26.7%)	1216 (25.77%)

35-39	6 (20.0%)	989 (20.96%)

40+	3 (10.0%)	604 (12.80%)

**Household asset group (at baseline)**		

Poorest	18 (60.0%)	1433 (30.4%)

Middle	10 (33.3%)	1258 (26.7%)

Richest	1 (3.3%)	1778 (37.7%)

Missing	1 (3.3%)	250 (5.3%)

**Mother’s educational attainment (at baseline)**		

No schooling	13 (43.3%)	2680 (56.8%)

Grades 1-5	1 (3.3%)	506 (10.7%)

Grades 6-9	1 (3.3%)	211 (4.5%)

Beyond grade 9	1 (3.3%)	140 (3.0%)

Missing	14 (46.7%)	1182 (25.1%)

**Table 3:** Characteristics of children in the Butajira cohort, 1987-2011

	Maternal death (42 days)		Mother survived
	**Index children**	**Non-index children**	**Children**

n	32	72	16875

**Sex of child**			

Boy	14 (43.8%)	38 (52.8%)	8679 (51.4%)

Girl	18 (56.2%)	34 (47.2%)	8196 (48.6%)

**Birth order**			

1	11 (34.4%)	18 (25.0%)	4719 (28.0%)

2	4 (12.5%)	18 (25.0%)	3614 (21.4%)

3	0	16 (22.2%)	2803 (16.6%)

4	5 (15.6%)	8 (11.1%)	2077 (12.3%)

5+	12 (37.5%)	12 (16.7%)	3662 (21.7%)

In data presented in Additional File 1, children who lost a mother (from any cause, maternal or not) were significantly more likely to have received no schooling: 55% of children with surviving mothers never attended school, versus 62% of children with deceased mothers (p-value <0.01). Note that, unlike vital and demographic events, education was not updated in the routine surveillance system, so this variable is often missing in the dataset and these data should be interpreted with caution. Additionally, cause of death is also largely missing (for two-thirds of deceased children) so differences between groups are too small to be analyzed—but, among all child deaths, the most common attributed causes included stillbirths, diarrhea/vomiting, sudden death, pneumonia, malaria, and malnutrition (presented in Additional File 2).

**Figure 1:**
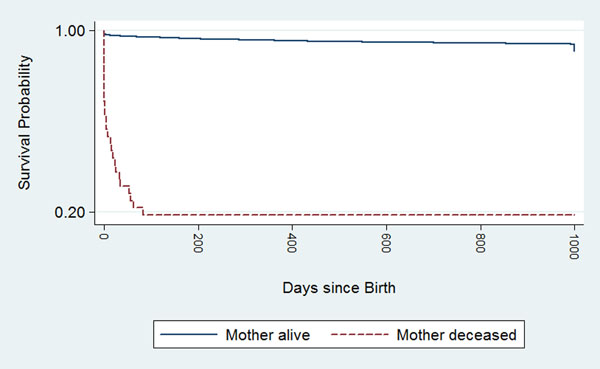
Kaplan-Meier Survival Probability Curve by maternal mortality status in the Butajira cohort, 1987-2011

Maternal death had a large and significant impact on child survival, as shown in Table 4 and Figure 1. (These survival analysis results, and all that follow, compare index children from maternal deaths to children whose mothers survived.) All deaths among index children of women who died during or after childbirth occurred in the first year of life. These survival functions are significantly different (log-rank test p<0.001). Data from Table 4 are presented visually in a Kaplan-Meier survival curve, seen in Figure 1. The survival function for non-index children of maternal deaths (see Additional File 3) is not significantly different from that of their counterparts with surviving mothers (p=0.38)—indicating that the mortality effect of a maternal death was strongest among the index children, and, as seen in Table 4 and Figure 1, was concentrated during early infancy.

**Table 4:** Probability of survival to day x for index children by maternal mortality status, in the Butajira cohort, 1987-2011

	Maternal death		Mother survived	
Days since birth	Survival prob.	n died	Survival prob.	n died

0	0.6875	10	0.9848	274

30	0.3750	10	0.9760	159

183	0.1875	6	0.9638	218

365	0.1875	0	0.9558	142

1825	0. 1875	0	0.9279	463

3652	0. 1875	0	0.9167	152

Table 5 presents age-specific mortality rates by mother survival status: the death rate from birth to 30 days was 81.92 per 100,000 child-days for children of surviving mothers, and 4210.53 deaths per 100,000 child-days for index children of maternal deaths. Thus, index children of maternal deaths were at 46 times greater risk of dying in the first month of life than their counterparts whose mothers survived; or, if adjusting for covariates, a 57 times greater risk of dying during the first 30 days of life.

**Table 5:** Age specific death rates in children according to survival status of the mother in the Butajira cohort, 1987-2011

Child age (days)	Deaths per 100000 child-days (Number of child deaths)		Crude death rate ratio (95% CI)	Adjusted death rate ratio (95% CI)
			
	Mother survived	Maternal deaths (index children)		
0-30	81.92 (433)	4210.53 (20)	46.01 (25.84-81.92)	57.24 (25.31-129.49)

30-183	8.17 (218)	565.50 (6)	65.96 (24.94-174.41)	80.38 (21.93-294.59)

183-365	4.58 (142)			

365-730	2.94 (176)			

730-1095	1.73 (99)			

Note: Variables used for adjusted ratios include: household wealth, mother’s age, mother’s ever marital status, and mother’s educational attainment.

In Table 6, we explore the potential for gender bias in child mortality rates by maternal survival status. Unadjusted ratios during the neonatal period were higher for index boys than for index girls (though confidence intervals overlap so these may not be statistically significantly different), which corresponds to the widespread generally higher mortality risk faced by infant boys.

**Table 6:** Gender bias in child survival in the Butajira cohort, 1987-2011

Child age (days)	Deaths per 100000 child days (number of child deaths)		Crude death rate ratio (95% CI)	Adjusted death rate ratio (95% CI)
			
	Mother survived	Maternal deaths (index children)		
0-30				

Male	95.59 (259)	11111.11 (12)	101.93 (43.03-241.46)	79.46 (33.62-187.82)

Female	67.54 (174)	2179.84 (8)	29.06 (13.36-63.20)	40.42 (9.37-174.42)

30-183				

Male	7.97 (109)	555.56 (1)	64.42 (6.50-638.64)	56.48 (5.10-625.39)

Female	8.38 (109)	567.54 (5)	64.92 (22.08-190.90)	64.81 (14.31-293.47)

183-365				

Male	4.53 (72)			

Female	4.60 (70)			

Note: Variables used for adjusted ratios include: household wealth, mother’s age, mother’s ever marital status, and mother’s educational attainment.

Additionally, we explored the robustness of these results to an expanded definition of maternal death, up to 365 days postpartum (full results presented in Additional Files 4, 5, 6 and 7). Among the 58 women who died in the year after childbirth (which, by definition, includes the women captured in the 42-day definition of maternal mortality), 38 (or 63%) of their index children experienced a subsequent death. The survival function for index children of these women was significantly different than that for children of surviving mothers (p<0.001). The one-month mortality rate ratio for these children versus those born to mothers who survived was 15.2 (95% confidence interval 8.96-25.74), or adjusted ratio of 19.42 (9.24-40.85); and for the 30-183 day period, the ratio was 24.94 (13.76-45.18), or adjusted ratio of 27.96 (11.11-70.39). Thus, even with an expanded definition of maternal mortality that includes deaths up to 365 days after childbirth, these children also experience greatly increased likelihood of dying.

## Discussion

These results indicate a very high likelihood of infant mortality subsequent to a maternal death during the intra- and post-partum period. Children whose mothers died during or shortly after childbirth were at approximately 50 times greater risk of dying during the first month of life than babies whose mothers survived. A significantly elevated risk ratio was also found for children born to women who died within one year of childbirth. The results remained significant with the inclusion of control variables that might also be associated with higher mortality for both mother and child (maternal age, household wealth, maternal educational attainment)—indicating that these babies’ risk was likely linked to the maternal death itself and not other covariates.

This highly elevated risk of death for infants of maternal deaths echoes, and even surpasses, findings from other settings [6]. Local characteristics around childbirth and infant care, as well as household structure, may offer hypotheses to help explain the added risk seen here. In a study of childbearing practices in Butajira, the overwhelming majority of women (90%) gave birth at home, not at a health facility, and just one-quarter of deliveries were attended by a skilled provider [20]. Low use of clinical obstetric care may put both mothers and babies at increased risk of death from childbirth-related complications. This same study in Butajira also found that breastfeeding was initiated within one hour of birth for one-third of women, and almost all infants (99.6%) were still breastfed at one year of age [20]; in this environment of prompt, near-universal and long-duration breastfeeding, a maternal death that compromises the availability of breast milk could have especially dire nutritional consequences for babies. A qualitative study of the consequences of maternal death in Butajira found that most deaths among orphaned neonates occurred following a nutritional deficiency due to loss of breastfeeding, since formula was reportedly unaffordable; this study also reported poorer health care for orphaned children (e.g., lower rates of immunizations and medical careseeking when ill) [21]. Additionally, migratory patterns and household composition may leave orphaned infants at higher risk of death: male migration to urban centers, including Addis Ababa, is common in the study area, with outmigration incidence rate among males of 4.16 per 100 person years (95% confidence interval 4.10-4.22) [unpublished data]. If a mother dies, there is therefore a higher likelihood of decreased care and support in the household, which may contribute to the higher risk of child death in this group. Without formal social support mechanisms to assist families who suddenly find themselves caring for orphaned children (e.g., nutritional support, assistance with education and health care), the level of care will vary based on what these guardians can provide, creating possible additional vulnerabilities for orphaned children. These themes were echoed in the above-mentioned qualitative analysis in Butajira, where respondents discussed how fathers rarely stepped into the role of primary caregiver of their children following the loss of the mother, and how extended families (grandparents, and sometimes step-mothers) sometimes took on this role but faced myriad challenges [21]. That study also noted a lack of structured support mechanisms for orphan care.

Many women, and consequently children, could be saved by increasing availability and use of emergency obstetric and neonatal care. Because many obstetric complications arise unexpectedly, all women should be provided access to skilled attendants and emergency obstetric care. Interventions may need to target relevant barriers to use, including financial and time costs, accessibility issues (transportation availability), quality of care (including clinical indicators and treatment by staff), and psychic costs and social norms that may be limiting utilization of maternity care. It has been acknowledged that maximum impact requires pairing clinical care with community-based interventions [22], which is particularly important in a context such as Butajira where few women currently have institutional deliveries. Programs to address upstream risk factors for maternal death, including women’s economic empowerment and increased female educational attainment [23], are likewise also important for child well-being. Although this study did not find mortality effects for the older children orphaned by a maternal death, the much lower rate of any educational attainment in this group indicates a potentially grave problem around orphan care and well-being. This also may perpetuate the cycle of poverty (and accordingly, the risk of maternal and infant mortality) from one generation to the next.

This study utilized the unique benefits of a large longitudinal dataset, with a high frequency of data collection over a long period of time, to examine the survival trajectories of children born to women who die during or shortly after childbirth. There are nonetheless some data limitations to note. First, there were ultimately few maternal deaths to analyze using either the 42- or 365-day cutoff values; this may limit the level of inference drawn from these analyses, and resulted in large confidence intervals around the results. Additionally, there are other possible confounding variables that could not be included in this analysis due to data availability. A third possible limitation is the use of a date-based method to code maternal deaths, which is subject to measurement error due to recall bias and misclassification of deaths from other causes that occurred during the postpartum period. In the absence of a vital registry in Butajira, verbal autopsy methods could offer a more specific approach to classifying maternal deaths; but the Butajira HDSS site only recently began using verbal autopsy methods so these data were not included here. To match the full WHO definition of maternal death (i.e., occurring during pregnancy or up to 42 days postpartum), the analysis was repeated, adding women who were reportedly pregnant at the time of their death. These results are included in Additional File 8; the survival function of children with mothers who died during pregnancy or within 42 days (there were 48 such women) was significantly different than that of children born to women who survived (p<0.001).

Finally, the generalizability of these results should be carefully considered. Contextual factors that may be crucially important for explaining the dramatic survival effects shown here likely vary by setting—so the results should be generalized with caution. For example, in settings with higher rates of institutional delivery and where high-quality neonatal and postnatal care is offered, these results may not hold. Additionally, differing practices around infant care (feeding behaviours, for example, and social networks for care) may be protective against infant death even following the loss of a mother. Lastly, there may be underlying risk factors that could detrimentally affect both maternal and infant survival—HIV or anemia, for example—and although data on those factors were not available in this dataset, such background risk is likely important and setting-dependent.

There are several important areas for follow-up research identified by this analysis. First, more quantified evidence is needed from a variety of settings on the survival of children following a maternal death. Second, there is much to be learned about non-mortality outcomes—for example educational attainment, morbidities (for example, nutritional status) and household structure—for surviving orphans. Lastly, robust evaluation data are lacking on effective ways to improve child survival following death of a mother, with respect to both health system approaches and family-community interventions.

## Conclusions

Saving mothers means saving children in this study context—a particularly salient finding given the slow progress in reducing neonatal mortality worldwide, which is seeing all too little improvement in Sub-Saharan Africa and constitutes approximately 40% of all under-5 deaths [24]. Infants of women who died during or shortly after childbirth were at significantly and dramatically elevated risk of death when compared to babies whose mothers survived; this relationship persists even after controlling for covariates. There appear also to be ill consequences for older orphaned children, notably in lesser educational attainment. The social and economic factors that undergird high maternal mortality rates also affect babies, both directly through the loss of a mother as well as indirectly over the life course. This study adds evidence—which has been noted as generally lacking [23]—on the inter-generational consequences of maternal death. It highlights the urgent need to scale up clinical programs for safe childbirth and postpartum care that also meet the needs of newborns, as well as to eliminate the barriers to women’s effectively utilizing such services. These results show that a maternal death has grave spillover effects, and the relationship between maternal and infant mortality merits further attention by policymakers and researchers.

## Competing interests

The authors declare that they have no competing interests.

## Authors' contributions

CM participated in study design and planning, performed the statistical analyses, and drafted the manuscript. AW participated in study conception and design, in the analyses, and in manuscript edits. MM participated in study conception, and in manuscript edits. JEF participated in study design and planning, and in manuscript edits. JL participated in study conception and design. AEY participated in participated in study conception and design, and in manuscript edits. All authors read and approved the final manuscript.

## Peer review

Reviewer reports for this article can be found in Additional file 9.

## Supplementary Material

Additional file 1 Supplementary Table 1: Education attainment of children in the Butajira cohort, by mother survival status (excludes children with missing value for education variable), 1987-2011 Click here for file

Additional file 2 Supplementary Table 2: Cause of death among deceased children in the Butajira cohort (excludes children with missing value for cause of death), 1987-2011 Click here for file

Additional file 3 Supplementary Table 3: Probability of survival to day x for non-index children by maternal mortality status, in Butajira cohort, 1987-2011 Click here for file

Additional file 4 Supplementary Table 4: Characteristics of mothers and children in the Butajira cohort, expanded definition for late maternal death, 1987-2011 Click here for file

Additional file 5 Supplementary Table 5: Probability of survival to day x for index children by maternal mortality status, expanded definition for late maternal death in Butajira cohort, 1987-2011 Click here for file

Additional file 6 Supplementary Figure 1: Kaplan-Meier Survival Probability Curve by maternal mortality status, expanded definition for late maternal death in Butajira cohort, 1987-2011 Click here for file

Additional file 7 Supplementary Table 6: Age specific death rates in children according to survival status of the mother, expanded definition for late maternal death in Butajira cohort, 1987-2011 Click here for file

Additional file 8 Supplementary Table 7: Probability of survival to day x for index children by maternal mortality status, maternal deaths include those during pregnancy and up to 42 days postpartum in Butajira cohort, 1987-2011 Click here for file

Additional file 9 Click here for file

## Declarations

This article has been published as part of *Reproductive Health* Volume 12 Supplement 1, 2015: True costs of maternal death. The full contents of the supplement are available online at http://www.reproductive-health-journal.com/supplements/12/S1. Publication charges for this supplement were funded by Family Care International and the FXB Center for Health and Human Rights.
