# Supplementary material for: Consequences of maternal mortality on infant and child survival: a 25-year longitudinal analysis in Butajira Ethiopia (1987-2011)
Source: Reprod Health. 2015 May 6;12(Suppl 1):S4. doi: 10.1186/1742-4755-12-S1-S4 (PMC4423767; doi:10.1186/1742-4755-12-S1-S4)
Supplement: Additional file 1 — Supplementary Table 1: Education attainment of children in the Butajira cohort, by mother survival status (excludes children with missing value for education variable), 1987-2011 [file 1742-4755-12-S1-S4-S1.pdf]

Supplementary Table 1: Education attainment of children in the Butajira cohort, by mother survival status (excludes children with missing value for education variable), 1987-2011

	**Mother deceased**	**Mother survived**
n	557	5028
No schooling	2784 (55.4%)	348 (62.5%)
Grades 1-5	1529 (30.4%)	168 (30.2%)
Grades 6-8	405 (8.1%)	26 (4.7%)
Grades 9+	310 (6.2%)	15 (2.7%)
