# Supplementary material for: Consequences of maternal mortality on infant and child survival: a 25-year longitudinal analysis in Butajira Ethiopia (1987-2011)
Source: Reprod Health. 2015 May 6;12(Suppl 1):S4. doi: 10.1186/1742-4755-12-S1-S4 (PMC4423767; doi:10.1186/1742-4755-12-S1-S4)
Supplement: Additional file 2 — Supplementary Table 2: Cause of death among deceased children in the Butajira cohort (excludes children with missing value for cause of death), 1987-2011 [file 1742-4755-12-S1-S4-S2.pdf]

Supplementary Table 2: Cause of death among deceased children in the Butajira cohort (excludes children with missing value for cause of death), 1987-2011

Cause	n
Stillbirth	122
Diarrhea/vomiting	103
Sudden death	61
Pneumonia	61
Malaria	54
Malnutrition	54
Accident	27
Tuberculosis	21
Premature birth	14
Whooping cough	14
Hepatitis	9
Pregnancy/ delivery related	5
Measles	2
Meningitis	1
AIDS	1
Other	310
