# Supplementary material for: Consequences of maternal mortality on infant and child survival: a 25-year longitudinal analysis in Butajira Ethiopia (1987-2011)
Source: Reprod Health. 2015 May 6;12(Suppl 1):S4. doi: 10.1186/1742-4755-12-S1-S4 (PMC4423767; doi:10.1186/1742-4755-12-S1-S4)
Supplement: Additional file 3 — Supplementary Table 3: Probability of survival to day x for non-index children by maternal mortality status, in Butajira cohort, 1987-2011 [file 1742-4755-12-S1-S4-S3.pdf]

Supplementary Table 3: Probability of survival to day x for non-index children by maternal mortality status, in Butajira cohort, 1987-2011

	**Maternal death**		**Mother survived**	
Days since birth	Survival prob.	n died	Survival prob.	n died
0	1.0	0	0.9847	274
30	1.0	0	0.9759	159
183	1.0	0	0.9637	218
365	1.0	0	0.9557	142
1825	0.9861	1	0.9276	462
3652	0.9716	1	0.9165	151
