# Supplementary material for: Consequences of maternal mortality on infant and child survival: a 25-year longitudinal analysis in Butajira Ethiopia (1987-2011)
Source: Reprod Health. 2015 May 6;12(Suppl 1):S4. doi: 10.1186/1742-4755-12-S1-S4 (PMC4423767; doi:10.1186/1742-4755-12-S1-S4)
Supplement: Additional file 4 — Supplementary Table 4: Characteristics of mothers and children in the Butajira cohort, expanded definition for late maternal death, 1987-2011 [file 1742-4755-12-S1-S4-S4.pdf]

Supplementary Table 4: Characteristics of mothers and children in the Butajira cohort, expanded definition for late maternal death, 1987-2011

			n	%
**Maternal death within 365 days** n= 58	Index child n= 60	Deceased	38	63.33%
		Survived	22	36.67%
	Non-index children n= 151	Deceased	7	4.64%
		Survived	144	95.36%
**Non-maternal death** n= 298	Children n= 907	Deceased	31	3.42%
		Survived	876	95.58%
**Surviving women** n= 4719	Children n= 16875	Deceased	1509	8.94%
		Survived	15366	91.06%
