# Supplementary material for: Consequences of maternal mortality on infant and child survival: a 25-year longitudinal analysis in Butajira Ethiopia (1987-2011)
Source: Reprod Health. 2015 May 6;12(Suppl 1):S4. doi: 10.1186/1742-4755-12-S1-S4 (PMC4423767; doi:10.1186/1742-4755-12-S1-S4)
Supplement: Additional file 5 — Supplementary Table 5: Probability of survival to day x for index children by maternal mortality status, expanded definition for late maternal death in Butajira cohort, 1987-2011 [file 1742-4755-12-S1-S4-S5.pdf]

Supplementary Table 5: Probability of survival to day x for index children by maternal mortality status, expanded definition for late maternal death in Butajira cohort, 1987-2011

	**Maternal death**		**Mother survived**	
Days since birth	Survival prob.	n died	Survival prob.	n died
0	0.8333	10	0.9848	274
30	0.6667	10	0.9759	159
183	0.4667	12	0.9641	212
365	0.3833	5	0.9564	137
1825	0.3667	1	0.9284	462
3652	0.3667	0	0.9173	152
