# Supplementary figures and images for: Consequences of maternal mortality on infant and child survival: a 25-year longitudinal analysis in Butajira Ethiopia (1987-2011)
Source: Reprod Health. 2015 May 6;12(Suppl 1):S4. doi: 10.1186/1742-4755-12-S1-S4 (PMC4423767; doi:10.1186/1742-4755-12-S1-S4)

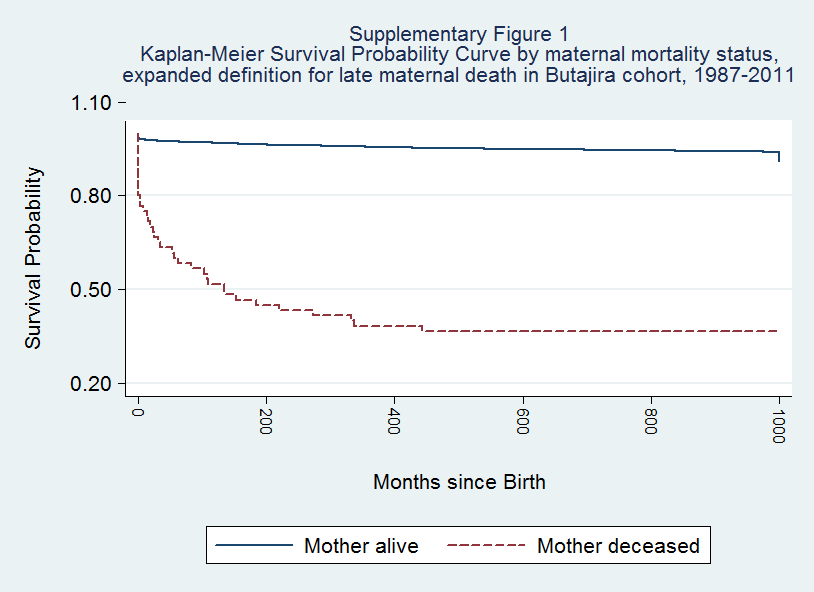

Supplement: Additional file 6 — Supplementary Figure 1: Kaplan-Meier Survival Probability Curve by maternal mortality status, expanded definition for late maternal death in Butajira cohort, 1987-2011 [file 1742-4755-12-S1-S4-S6.tif]
