# Supplementary material for: Consequences of maternal mortality on infant and child survival: a 25-year longitudinal analysis in Butajira Ethiopia (1987-2011)
Source: Reprod Health. 2015 May 6;12(Suppl 1):S4. doi: 10.1186/1742-4755-12-S1-S4 (PMC4423767; doi:10.1186/1742-4755-12-S1-S4)
Supplement: Additional file 7 — Supplementary Table 6: Age specific death rates in children according to survival status of the mother, expanded definition for late maternal death in Butajira cohort, 1987-2011 [file 1742-4755-12-S1-S4-S7.pdf]

Supplementary Table 6: Age specific death rates in children according to survival status of the mother, expanded definition for late maternal death in Butajira cohort, 1987-2011

**Child age (days)**	**Deaths per 100000 child-days (Number of child deaths)**		**Crude death rate ratio (95% CI)**	**Adjusted death rate ratio (95% CI)**
	Mother survived	Maternal deaths (index children)		
0-30	82.05 (433)	1520.91 (20)	15.20 (8.96-25.74)	19.42 (9.24-40.85)
30-183	7.96 (212)	240.48 (12)	24.94 (13.76-45.18)	27.96 (11.11-70.39)
183-365	4.42 (137)	108.27 (5)	22.30 (9.14-54.39)	19.47 (4.85-78.18)
365-730	2.92 (175)	12.52 (1)	3.11 (0.43-22.69)	0 (0-0)
730-1095	1.74 (99)	.	.	.
