# Supplementary material for: Consequences of maternal mortality on infant and child survival: a 25-year longitudinal analysis in Butajira Ethiopia (1987-2011)
Source: Reprod Health. 2015 May 6;12(Suppl 1):S4. doi: 10.1186/1742-4755-12-S1-S4 (PMC4423767; doi:10.1186/1742-4755-12-S1-S4)
Supplement: Additional file 8 — Supplementary Table 7: Probability of survival to day x for index children by maternal mortality status, maternal deaths include those during pregnancy and up to 42 days postpartum in Butajira cohort, 1987-2011 [file 1742-4755-12-S1-S4-S8.pdf]

Supplementary Table 7: Probability of survival to day x for index children by maternal mortality status, maternal deaths include those during pregnancy and up to 42 days postpartum in Butajira cohort, 1987-2011

	**Maternal death**		**Mother survived**	
Days since birth	Survival prob.	n died	Survival prob.	n died
0	0.9333	10	0.9847	274
30	0.8667	10	0.9758	159
183	0.8267	6	0.9636	218
365	0.8267	0	0.9555	142
1825	0.8199	1	0.9274	462
3652	0.8053	2	0.9163	150
