# Supplementary material for: Consequences of maternal mortality on infant and child survival: a 25-year longitudinal analysis in Butajira Ethiopia (1987-2011)
Source: Reprod Health. 2015 May 6;12(Suppl 1):S4. doi: 10.1186/1742-4755-12-S1-S4 (PMC4423767; doi:10.1186/1742-4755-12-S1-S4)
Supplement: Additional file 9 [file 1742-4755-12-S1-S4-S9.pdf]

Referee's comments to the authors– this sheet WILL be seen by the author(s) and published with the article

Title	Consequences of maternal mortality on infant and child survival: a 25-year longitudinal analysis in Butajira Ethiopia (1987-2011)
Author(s)	Corrina Moucheraud, Alemayehu Worku, Mitike Molla, Jocelyn E Finlay, Jennifer Leaning, Alicia Ely Yamin
Referee's name	France Donnay

When assessing the work, please consider the following points, where applicable:

1. Is the question posed by the authors new and well defined?
2. Are the methods appropriate and well described, and are sufficient details provided to replicate the work?
3. Are the data sound and well controlled?
4. Does the manuscript adhere to the relevant standards for reporting and data deposition?
5. Are the discussion and conclusions well balanced and adequately supported by the data?
6. Do the title and abstract accurately convey what has been found?
7. Is the writing acceptable?

Please make your report as constructive and detailed as possible in your comments so that authors have the opportunity to overcome any serious deficiencies that you find and please also divide your comments into the following categories:

- Major Compulsory Revisions (which the author must respond to before a decision on publication can be reached)
- Minor Essential Revisions (such as missing labels on figures, or the wrong use of a term, which the author can be trusted to correct)
- Discretionary Revisions (which are recommendations for improvement but which the author can choose to ignore)

Where possible please supply references to substantiate your comments.

When referring to the manuscript please provide specific page and paragraph citations where appropriate.

General comments: This is a very interesting and timely paper presenting additional evidence on the impact of maternal deaths on young infants. The need to ensure quality care at childbirth for both mother and newborn is reaffirmed at a time when a development framework for 2015 – 2030 is being discussed. Previous studies in Bangladesh and several countries in Africa are described and this study is a useful addition to the body of evidence, given the unique opportunity to follow a population cohort during a long period. Morbidity was included, making the case even more compelling.

One can think of a similar study replicated in a setting with a high rate of institutional deliveries ? urban or periurban settings where feeding patterns would be different ? what is the role of social networks in these circumstances? Minor revisions could include a discussion on urban/rural difference and the role of social networks.

Finally, a new finding – for me – was the high survival rate of non index children, even if access to education was deeply affected by their mother 's death.

Major compulsory revisions: none

(continue on the next sheet)

Referee's comments to the authors– this sheet WILL be seen by the author(s) and published with the article

Title	Consequences of maternal mortality on infant and child survival: a 25-year longitudinal analysis in Butajira Ethiopia (1987-2011)
Author(s)	Corrina Moucheraud, Alemayehu Worku, Mitike Molla, Jocelyn E Finlay, Jennifer Leaning, Alicia Ely Yamin
Referee's name	Michel Garenne

When assessing the work, please consider the following points, where applicable:

1. Is the question posed by the authors new and well defined?
2. Are the methods appropriate and well described, and are sufficient details provided to replicate the work?
3. Are the data sound and well controlled?
4. Does the manuscript adhere to the relevant standards for reporting and data deposition?
5. Are the discussion and conclusions well balanced and adequately supported by the data?
6. Do the title and abstract accurately convey what has been found?
7. Is the writing acceptable?

Please make your report as constructive and detailed as possible in your comments so that authors have the opportunity to overcome any serious deficiencies that you find and please also divide your comments into the following categories:

- Major Compulsory Revisions (which the author must respond to before a decision on publication can be reached)
- Minor Essential Revisions (such as missing labels on figures, or the wrong use of a term, which the author can be trusted to correct)
- Discretionary Revisions (which are recommendations for improvement but which the author can choose to ignore)

Where possible please supply references to substantiate your comments.

When referring to the manuscript please provide specific page and paragraph citations where appropriate.

General comments (confidential to authors):

The paper presents an analysis of child survival after the death of a mother in a rural Ethiopian population under demographic surveillance. The material is important for public health purposes, and data quality seems high. However, the paper suffers from serious methodological problems, and deserves to be revised before it could be published.
Comments:
1. Case definition of maternal mortality: the authors do not follow international guidelines, and even though they mention this issue in the discussion, their presentation is often confusing. It seems that they want to focus on intra-partum and post-partum mortality and its consequences: why not calling it by its name?
2. Case definition of mortality of children: the authors seem to include stillbirths in their analysis. I suggest to distinguish properly the following cases: still birth / neonatal death / post-neonatal etc.; or the following: perinatal, other infant death etc.
3. Comparisons: it seems that authors want to compare the survival of babies who lost their mother during delivery or during the post-partum period (42 days) (called index children) with two types of controls: children of the same mother born before (non-index), and children of other mothers who survived. This should be said properly from the beginning, and tables should be presented this way. As an option (not recommended), they could also add a fourth category of children whose mothed died after the post-partum period and before a year after delivery. But this would require a separate survival analysis, since the starting point for survival is different.

(continue on the next sheet)

Continued:
Major compulsory revisions:

Minor essential revisions:

Discretionary revisions:

Commentary (for publishing)

The study of child survival after the death of a mother is important for public health purposes. Too many women and too many children still die from such causes, especially in Africa, while proper obstetric care and postnatal care available elsewhere could help preventing most of them. The study presents important material, accurate data, and impressive results: the death of a mother is almost synonymous to a very premature death of the baby. Of course, readers would like to know more on this population: what are the causes of death of mothers and children? What can be done about it? Why so many women still deliver at home? What were the traditional ways of handling orphans (child, care, breastfeeding, etc.)? Are they new programs to take care of orphans? Publishing this type of information should raise awareness about specific reproductive health issues, and should lead to improvements in the health situation of mothers and children in the long run.
